# An Integrated Analysis of Tumor Purity of Common Central Nervous System Tumors in Children Based on Machine Learning Methods

**DOI:** 10.3389/fgene.2021.707802

**Published:** 2021-12-03

**Authors:** Jian Yang, Jiajia Wang, Shuaiwei Tian, Qinhua Wang, Yang Zhao, Baocheng Wang, Liangliang Cao, Zhuangzhuang Liang, Heng Zhao, Hao Lian, Jie Ma

**Affiliations:** Department of Pediatric Neurosurgery, Xinhua Hospital Affiliated to Shanghai Jiao Tong University School of Medicine, Shanghai, China

**Keywords:** pediatric, central nervous system tumors, medulloblastoma, tumor purity, machine learning

## Abstract

**Background:** Tumor purity is defined as the proportion of cancer cells in the tumor tissue, and its effects on molecular genetics, the immune microenvironment, and the prognosis of children’s central nervous system (CNS) tumors are under-researched.

**Methods:** We applied random forest machine learning, the InfiniumPurify algorithm, and the ESTIMATE algorithm to estimate the tumor purity of every child’s CNS tumor sample in several published pediatric CNS tumor sample datasets from Gene Expression Omnibus (GEO), aiming to perform an integrated analysis on the tumor purity of children’s CNS tumors.

**Results:** Only the purity of CNS tumors in children based on the random forest (RF) machine learning method was normally distributed. In addition, the children’s CNS tumor purity was associated with primary clinical pathological and molecular indicators. Enrichment analysis of biological pathways related to the purity of medulloblastoma (MB) revealed some classical signaling pathways associated with MB biology and development-related pathways. According to the correlation analysis between MB purity and the immune microenvironment, three immune-related genes, namely, CD8A, CXCR2, and TNFRSF14, were negatively related to MB purity. In contrast, no significant correlation was detected between immunotherapy-associated markers, such as PD-1, PD-L1, and CTLA4; most infiltrating immune cells; and MB purity. In the tumor purity–related survival analysis of MB, ependymoma (EPN), and children’s high-grade glioma, we discovered a minor effect of tumor purity on the survival of the aforementioned pediatric patients with CNS tumors.

**Conclusion:** Our purity pediatric pan-CNS tumor analysis provides a deeper understanding and helps with the clinical management of pediatric CNS tumors.

## Introduction

As the most frequent solid tumors in children, pediatric tumors of the central nervous system (CNS) represent an array of molecularly and clinically diverse entities. The tumor microenvironment (TME) is a complicated milieu comprising many factors that promote and inhibit tumor growth, nutrients, chemokines, and the spectrum of non-tumor cells (e.g., immunocytes, fibroblasts, and endotheliocytes). Increasing evidence has revealed that the TME plays a pivotal role in tumorigenesis, tumor progression, and the response to therapy (Schreiber et al., 2011).

For the past few years, high-throughput techniques have been increasingly applied in the field of pediatric CNS tumors (Kumar et al., 2018). These techniques offer some new means for the clinical diagnosis, prognostic prediction, and precise classification of pediatric CNS tumors. Nevertheless, the surgically acquired tumor tissues used for high-throughput techniques are a mixture of both tumor cells and non-tumor tissues. The DNA and RNA extracted from such a mixture are from all of the cells involved, so the measurement result is a kind of mixed signal (Zheng et al., 2017). Such a sample mixture may bias the downstream analyses and thus could mask true biologically meaningful signals.

Tumor purity is defined as the proportion of tumor cells in tumor tissue. Some recent studies have reported the confounding effect of tumor purity on gene clustering, coexpression networks, molecular taxonomy, and tumor prognosis and microenvironment (Aran et al., 2015; Rhee et al., 2018). Currently, there are three main methods available for tumor purity estimation. The first is to estimate the tumor purity based on the pathological images of the tumor tissue by histopathological researchers and clinical pathologists. However, these results are subject to the observer’s proficiency and the pathological sensitivity of the tumor tissue (Zhang et al., 2017). The second way determines tumor purity by virtue of cell sorting–based techniques such as magnetic-activated cell sorting (Schmitz et al., 1994) and fluorescent-activated cell sorting (Basu et al., 2010). However, these methods demand high inputs of time, effort, and money and are therefore difficult to apply in large-scale studies.

More recently, with the development of high-throughput techniques and improved bioinformatics approaches, many purity estimation methods by computational methods have been developed, and they are based on transcriptome data, copy number variation data, DNA methylation data, or genetic mutation data. These methods include the random forest (RF) algorithm based on DNA methylation data (Capper et al., 2018), ESTIMATE based on gene expression data (Yoshihara et al., 2013), ABSOLUTE based on somatic copy number data (Carter et al., 2012), and InfiniumPurify based on DNA methylation data (Zheng et al., 2017).

The existing studies on tumor purity are limited to adult samples from the Cancer Genome Atlas, and little is known regarding the relationship between tumor purity and the clinicopathologic or genomic features in pediatric CNS tumors. In addition, the association between the purity and microenvironment of pediatric CNS tumors remains unclear. In this study, we used these major means of tumor purity estimation to infer tumor purity and sought to evaluate the impact of purity on pediatric CNS tumor prognosis, genetic profiling, and the immune microenvironment, which may deepen our understanding of pediatric CNS tumor biology and provide new insights into the clinical management of pediatric CNS tumors.

## Materials and Methods

### Data Collection

The data of children’s CNS tumors (e.g., medulloblastoma (MB), ependymoma (EPN), pilocytic astrocytoma, diffuse midline glioma, atypical teratoma/rhomboid tumor, and embryonal tumor with multilayered rosettes) used in this study were from Gene Expression Omnibus (GEO) and ArrayExpress. Supplementary Table S1 lists the general information about the datasets involved.

### Selection of an Adequate Algorithm for Purity Estimation of Common Pediatric CNS Tumors

Random forest (RF), InfiniumPurify, and ESTIMATE algorithms were used to estimate tumor purity. The RF model was established by training the DNA methylation data extracted from the panglioma dataset (795 samples of glioma) (Ceccarelli et al., 2016) in TCGA based on the ABSOLUTE algorithm (a direct purity estimation method) (Capper et al., 2018). We selected the optimal algorithm from the aforementioned three algorithms according to the distribution of purity in different datasets of common pediatric CNS tumors.

### Exploration of Biological Functions Related to Common Pediatric CNS Tumor Purity

We screened the genes that correlated with tumor purity by Pearson correlation analysis (Pearson |R| > 0.3). In total, 1,051 genes were eligible for Gene Ontology (GO) enrichment analysis and gene set enrichment analysis (GSEA) (Subramanian et al., 2005). Both GO analysis and GSEA were performed utilizing the R package “clusterProfiler.” In addition, the cases were split into high- and low-purity groups based on the median purity. By utilizing the R package “GSVA,” we performed gene set variation analysis (GSVA) of hallmark pathways between the high- and low-purity samples (Hänzelmann et al., 2013).

### Evaluation of the Relationship Between the Purity of Common Pediatric CNS Tumors and the Tumor Microenvironment

By applying CIBERSORT(Gentles et al., 2015), we scored 22 immune cell types for their relative abundance in pediatric CNS tumor samples. For any given sample, we computed the relationships between tumor purity and the relative proportions of the individual immune cell types. In addition, we also computed the associations between tumor purity and the relative fractions of 24 immune cell types by using single-sample gene set enrichment analysis (ssGSEA) (Bindea et al., 2013), as implemented in the R package “GSVA.” Finally, we determined the correlations between tumor purity and 14 immune-related genes (GZMA, PRF1, CD8A, PD-1, PD-L1, CTLA4, IDO1, CXCR2, TNFRSF14, TNFRSF18, CD247, LAG3, BTLA, and HAVCR2).

### Survival Analysis

For each type of pediatric CNS tumors, we divided the samples into high- and low-purity groups based on the optimal cutoff value generated by using the R package “survMisc.” Kaplan–Meier (K-M) curves were used to estimate the overall survival distribution.

### Statistical Analysis

R software version 3.4.4 was employed for all statistical analyses. *p* values for the associations between tumor purity and the immune microenvironment were computed utilizing Pearson correlation analyses, followed by multiple testing utilizing the Benjamini–Hochberg method. For all statistical analyses, *p* < 0.05 was considered statistically significant.

## Results

### Selection of the Most Adequate Algorithm for Estimating the Purity of Common Pediatric CNS Tumors

**FIGURE 1:**
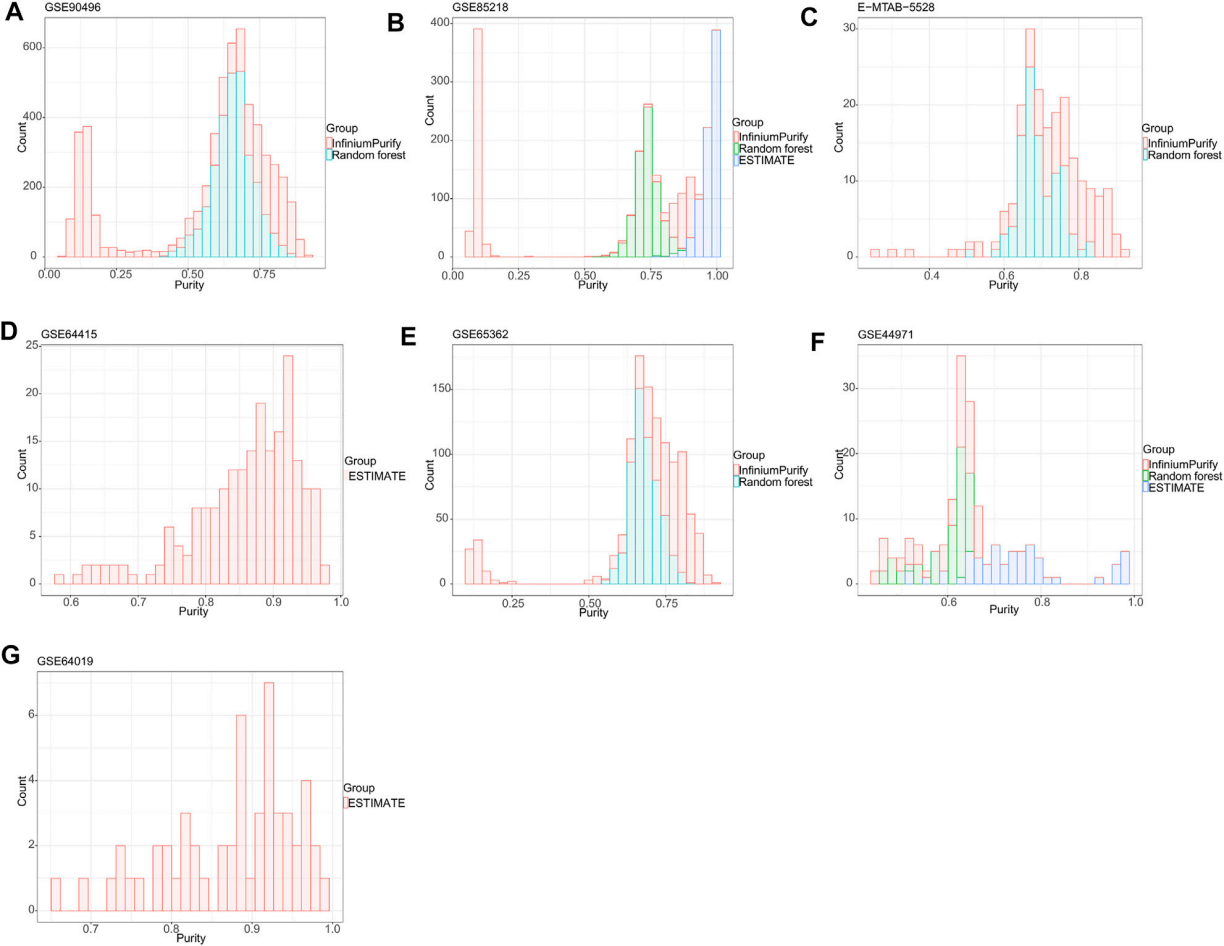
Tumor purity distribution of seven common pediatric CNS tumor datasets based on three methods of tumor purity estimation. **(A)** Tumor purity distribution of the GSE90496 dataset based on InfiniumPurify and random forest (RF) algorithms; **(B)** tumor purity distribution of the GSE85218 dataset based on the InfiniumPurify, ESTIMATE, and RF algorithms; **(C)** tumor purity distribution of the E-MTAB-5528 dataset based on the InfiniumPurify and RF algorithms; **(D)** tumor purity distribution of the GSE64415 dataset based on the ESTIMATE algorithm; **(E)** tumor purity distribution of the GSE65362 dataset based on the InfiniumPurify and RF algorithms; **(F)** tumor purity distribution of the GSE44971 dataset based on the InfiniumPurify, ESTIMATE, and RF algorithms; **(G)** tumor purity distribution of the GSE64019 dataset based on the ESTIMATE algorithm.

To establish a general understanding of the purity distribution of common pediatric CNS tumors, we estimated the tumor purity of samples in the GSE90496 datasets containing MB, EPN, pilocytic astrocytoma, diffuse midline glioma, atypical teratoma/rhomboid tumor, and embryonal tumor with multilayered rosettes. As shown in Figure 1A, the tumor purity distribution resulting from the InfiniumPurify algorithm had a bimodal pattern, with an average tumor purity of 49.8 ± 29.3%, while that from the RF algorithm was normal, with an average tumor purity of 65.9 ± 7.1%. Regarding the tumor purity distribution of the GSE85218 dataset (MB) (Figure 1B), the tumor purity based on the InfiniumPurify algorithm was bimodal (average tumor purity: 39.8 ± 37.6%), while that based on the ESTIMATE algorithm was skewed and focused on 80% or more of the total area (with an average tumor purity of 96.99 ± 3.3%), but the tumor purity resulting from the RF algorithm was normal, with an average tumor purity of 73.7 ± 4.5%. When applied to the E-MTAB-5528 dataset (diffuse midline glioma) (Figure 1C), the InfiniumPurify algorithm determined the tumor purity to be skewed and the average tumor purity to be 74.04 ± 12.4%, while the RF algorithm generated normal tumor purity, with an average value of 69.5 ± 5.5%. For the GSE64415 and GSE65362 datasets (EPN) (Figures 1D,E), the tumor purity based on the ESTIMATE algorithm was skewed, with an average value of 85.95 ± 8.01%, and that based on InfiniumPurify was also skewed, with an average value of 67.1 ± 22.4%, but that based on the RF algorithm was normal, with an average value of 68.4 ± 4.6%. For the GSE44971 dataset (pilocytic astrocytoma) (Figure 1F), the average tumor purities generated were 59.4 ± 6.9, 74.8 ± 11.8, and 59.9 ± 5.5% for InfiniumPurify, ESTIMATE, and RF, respectively, but they were all skewed. For the GSE64019 dataset (atypical teratoma/rhomboid tumor), the tumor purity distributed according to the ESTIMATE algorithm was skewed, with an average tumor purity of 87.4 ± 8.1%.

Judging from these results, the distribution of pediatric CNS tumors resulting from the ESTIMATE algorithm was skewed and focused on the part with over 70% of the total area, and the tumor purity distributions based on InfiniumPurify and RF were skewed and normal, respectively. The ESTIMATE method estimates purity indirectly by measuring stromal and immune counterparts in the tumor sample (Yoshihara et al., 2013). Therefore, the presence of non-stromal and immune cells in a cancer sample, such as contaminating adjacent normal cells, could affect ESTIMATE-based tumor purity estimation. In addition, the InfiniumPurify method estimates purity indirectly by identifying differentially methylated regions between cancer and normal samples (Zheng et al., 2017). However, paired normal controls were lacking in our pediatric pan–central nervous system tumor analysis. Although the InfiniumPurify method has a control-free variant, this is only applicable for tumor entities that are included in the TCGA datasets and not suitable for entities from the pediatric spectrum that we have used here. In contrast to the ESTIMATE and InfiniumPurify purity estimates, ABSOLUTE is a direct measure of the cancer cells in a sample (Carter et al., 2012). Taken together, we selected the ABSOLUTE-based RF method for the purity estimation of pediatric CNS tumors in this study, and all subsequent studies were based on the RF algorithm.

### Tumor Purity and Molecular and Clinicopathologic Features

**FIGURE 2:**
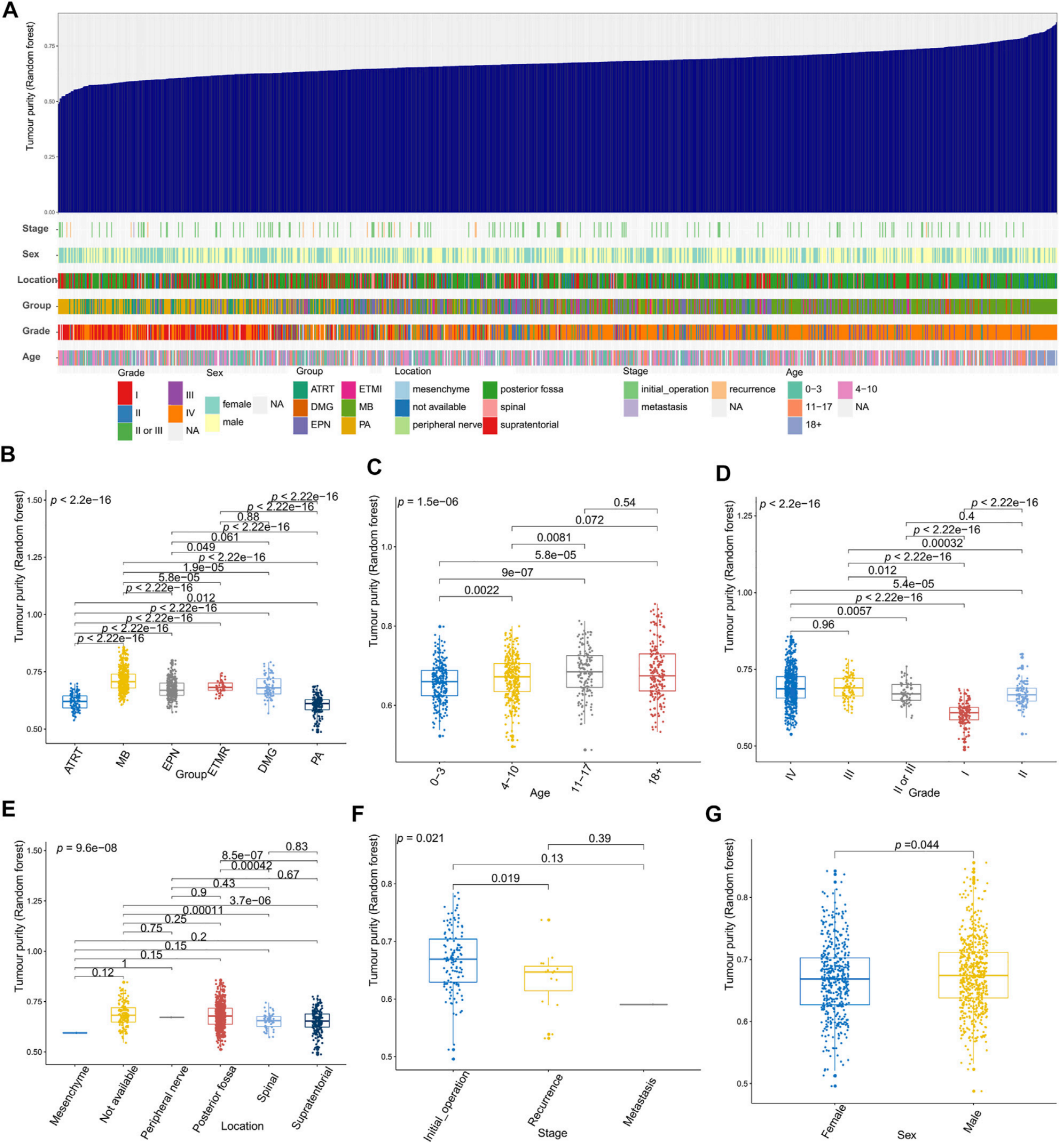
Relationship between tumor purity and the patients’ clinical features in the GSE90496 dataset. **(A)** An overview of the correlation between the clinical features and tumor purity in the GSE90496 dataset; **(B)** box plot of the tumor purity by tumor histology; **(C)** box plot of the tumor purity by age at diagnosis; **(D)** box plot of the tumor purity by the tumor grade; **(E)** Box plot of the tumor purity by the tumor location; **(F)** box plot of the tumor purity by the tumor grade; **(G)** box plot of the tumor purity by the patient gender.

**FIGURE 3:**
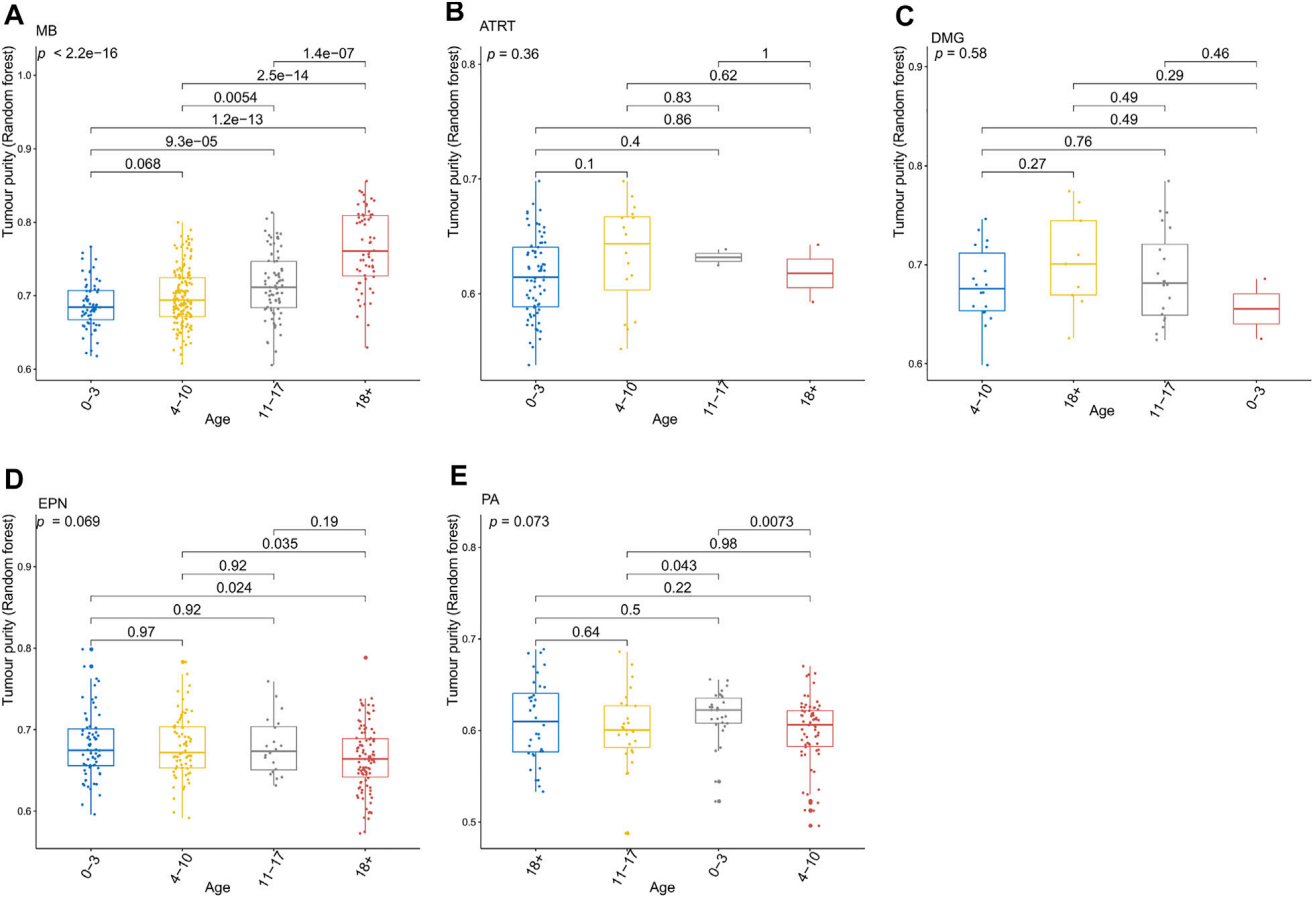
Relationship between the tumor purity of each pediatric CNS tumor and the patient age at diagnosis in the GSE90496 dataset. **(A)** Box plot of the tumor purity in medulloblastoma (MB) samples by the age at diagnosis; **(B)** box plot of the tumor purity in atypical teratoma/rhabdoid tumor samples by the age at diagnosis; **(C)** box plot of the tumor purity in diffuse midline glioma samples by the age at diagnosis; **(D)** box plot of the tumor purity in ependymoma (EPN) samples by the age at diagnosis; **(E)** box plot of the tumor purity in pilocytic astrocytoma samples by the age at diagnosis.

**FIGURE 4:**
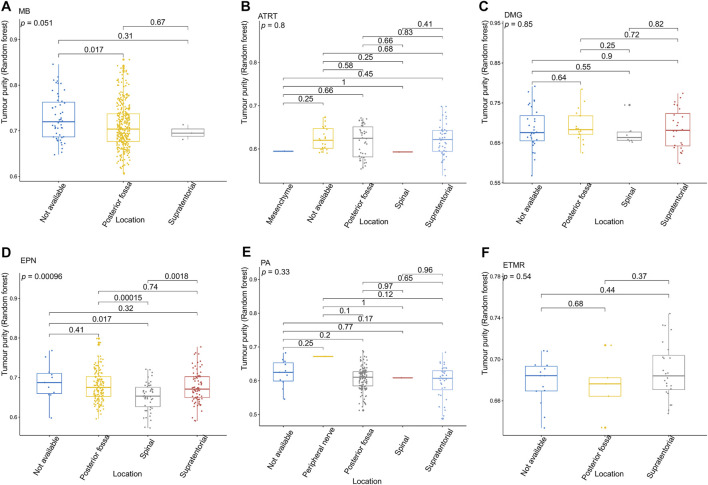
Relationship between the tumor purity of each pediatric CNS tumor and the tumor location in the GSE90496 dataset. **(A)** Box plot of the tumor purity in medulloblastoma (MB) samples by the tumor location; **(B)** box plot of the tumor purity in atypical teratoma/rhabdoid tumor samples by the tumor location; **(C)** box plot of the tumor purity in diffuse midline glioma samples by the tumor location; **(D)** box plot of the tumor purity in ependymoma (EPN) samples by the tumor location; **(E)** box plot of the tumor purity in pilocytic astrocytoma samples by the tumor location; **(F)** box plot of the tumor purity in embryonic tumors of multilayered rosettes by the tumor location.

Figures 2–4 illustrate the relationships between tumor purity and the patients’ clinical features in the GSE90496 dataset. For the tumor histology (Figures 2A,B), we observed that MB had the highest purity, whereas pilocytic astrocytoma and atypical teratoma/rhabdoid tumors had the lowest purity (*p* < 2.2e−16). For the age at diagnosis (Figures 2A,C), we found that the patients aged 0–3 years had the lowest tumor purity, while those older than 11 years had the highest purity (*p* = 1.5e−06). For the tumor grade (Figures 2A,D), the purity of Grade I tumor was the lowest, while that of Grade IV was the highest (*p* < 2.2e−16). For the tumor location (Figures 2A,E), we found that the purity of tumors located in the posterior cranial fossa was higher than that in the supratentorial parts (*p* = 8.5e−07). Regarding the tumor stage (Figures 2A,F), compared with primary tumors, recurrent tumors had lower purity (*p* = 0.019). For patient sex (Figures 2A,G), we observed higher tumor purity in male patients (*p* = 0.044) than in female patients.

**FIGURE 5:**
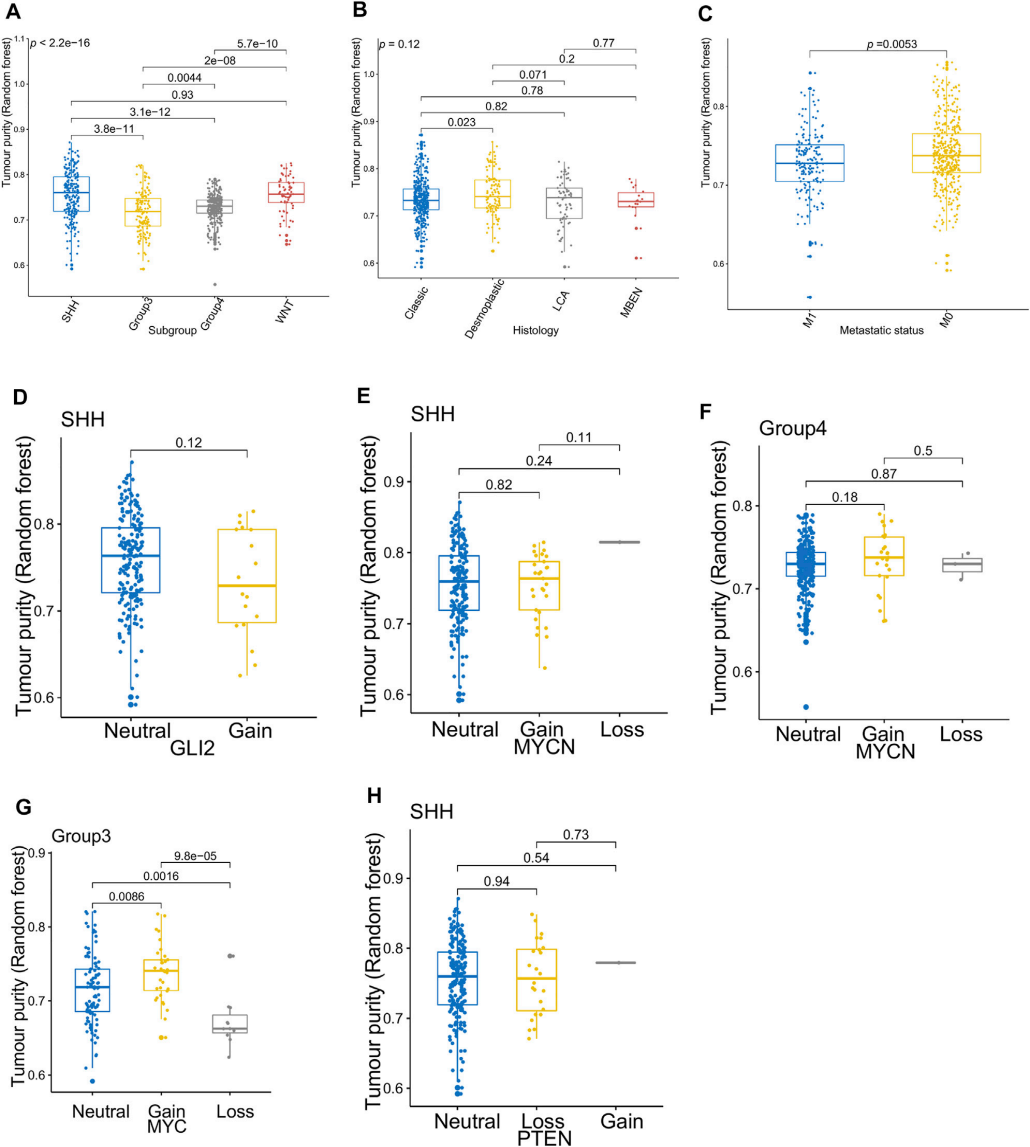
Relationship between the tumor purity of the medulloblastoma (MB) and its clinical molecular features in the GSE85218 dataset. **(A)** Box plot of the tumor purity by molecular subgroup; **(B)** box plot of the tumor purity by histopathology; **(C)** box plot of the tumor purity by metastatic status; **(D)** box plot of the tumor purity of the SHH subgroup by GLI amplification; **(E)** box plot of the tumor purity of the SHH subgroup by MYCN amplification; **(F)** box plot of the tumor purity of the Group 4 subgroup by MYCN amplification; **(G)** box plot of the tumor purity of the Group 3 subgroup by MYC amplification; **(H)** box plot of the tumor purity of the SHH subgroup by PTEN deletion.

Figure 3 presents the relationship between tumor purity and the age at diagnosis in each type of pediatric CNS tumor in the GSE90496 dataset. We found a positive correlation between tumor purity and the age at diagnosis in MB (*p* < 2.2e−16, Figure 3A) but not in other pediatric CNS tumors (including atypical teratoma/rhabdoid tumor, diffuse midline glioma, EPN, and pilocytic astrocytoma) (Figures 3B–E). As shown in Figures 4A–F, among six pediatric CNS tumors, no significant difference was detected between tumors located in the posterior cranial fossa and those in supratentorial sites in terms of tumor purity. The relationships between MB purity and clinicopathologic features in the GSE85218 dataset are shown in Figure 5. The four molecular subgroups of MB (Figure 5A) differed greatly from each other in terms of tumor purity (*p* < 2.2e−16). Compared with the non-WNT/SHH (Groups 3 and 4) subgroups of MB with an inferior prognosis, the WNT and SHH subgroups with a superior prognosis had a higher tumor purity. For the metastatic status of MB patients (Figure 5C), non-metastatic patients had higher tumor purity than metastatic patients (*p* = 0.0053). For the MYC amplifications of Group 3 MB patients (Figure 5G), the tumor purity of Group 3 MB with MYC amplifications was significantly different from that of Group 3 MB without MYC amplifications (MYC amplifications vs. MYC balance, *p* = 0.0086; MYC amplifications vs. MYC deletion, *p* = 9.8e-05). However, no significant difference was detected among all of the groups in tumor purity when other clinical and molecular features of MB were taken into account (Figures 5B,D–F,H).

**FIGURE 6:**
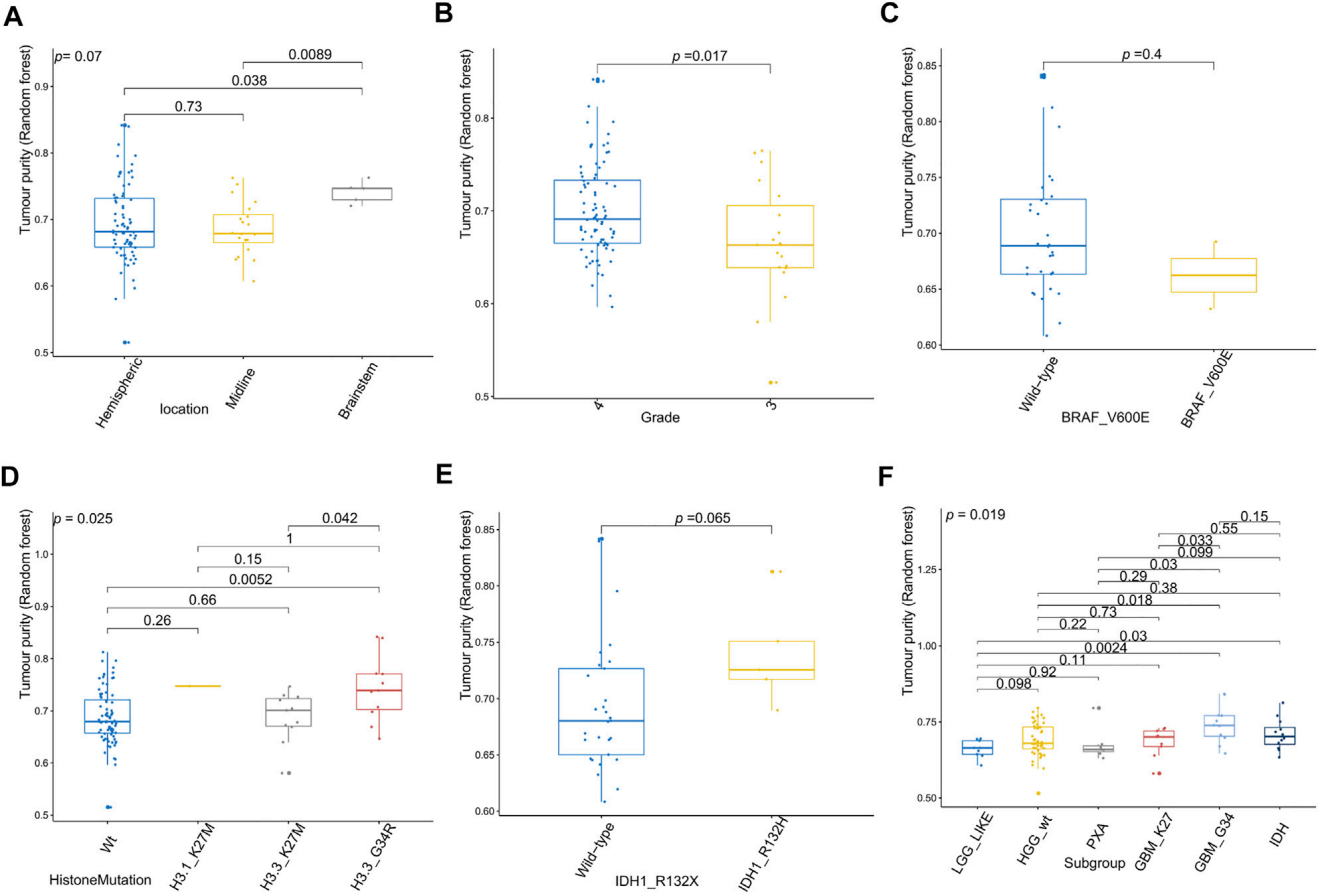
Relationship between tumor purity of high-grade glioma (HGG) and the patients’ clinical molecular features in the E-MTAB-5528 dataset. **(A)** Box plot of the tumor purity by tumor location; **(B)** box plot of the tumor purity by tumor grade; **(C)** box plot of the tumor purity by BRAF_V600E mutation status; **(D)** box plot of the tumor purity by histone mutation status; **(E)** box plot of the tumor purity by IDH1 mutation status; **(F)** box plot of the tumor purity by molecular subgroup.

Figure 6 shows the relationships between high-grade glioma tumor purity and the other clinicopathologic and molecular features in the E-MTAB-5528 dataset. However, for the tumor location (Figure 6A), none of the groups were significantly different from each other in tumor purity. For the tumor grade (Figure 6B), we found that the tumor purity of Grade IV patients was higher than that of Grade III patients (*p* = 0.017). Regarding BRAF_V600E mutation status (Figure 6C), no evident difference was found between the wild-type BRAF patients and mutant-type BRAF patients in tumor purity. For histone mutation status (Figure 6D), the tumor purity of subgroups divided by histone H3 mutation differed significantly (*p* = 0.025). For IDH1 mutation status (Figure 6H), patients with wild-type IDH1 were not significantly different from those with mutant-type IDH1 in tumor purity. Regarding the molecular subgroup (Figure 6F), a significant difference was detected between all of the molecular subgroups of high-grade glioma in tumor purity (*p* = 0.019).

### Functional Annotation of Transcriptomic Analysis in Tumor Purity

**FIGURE 7:**
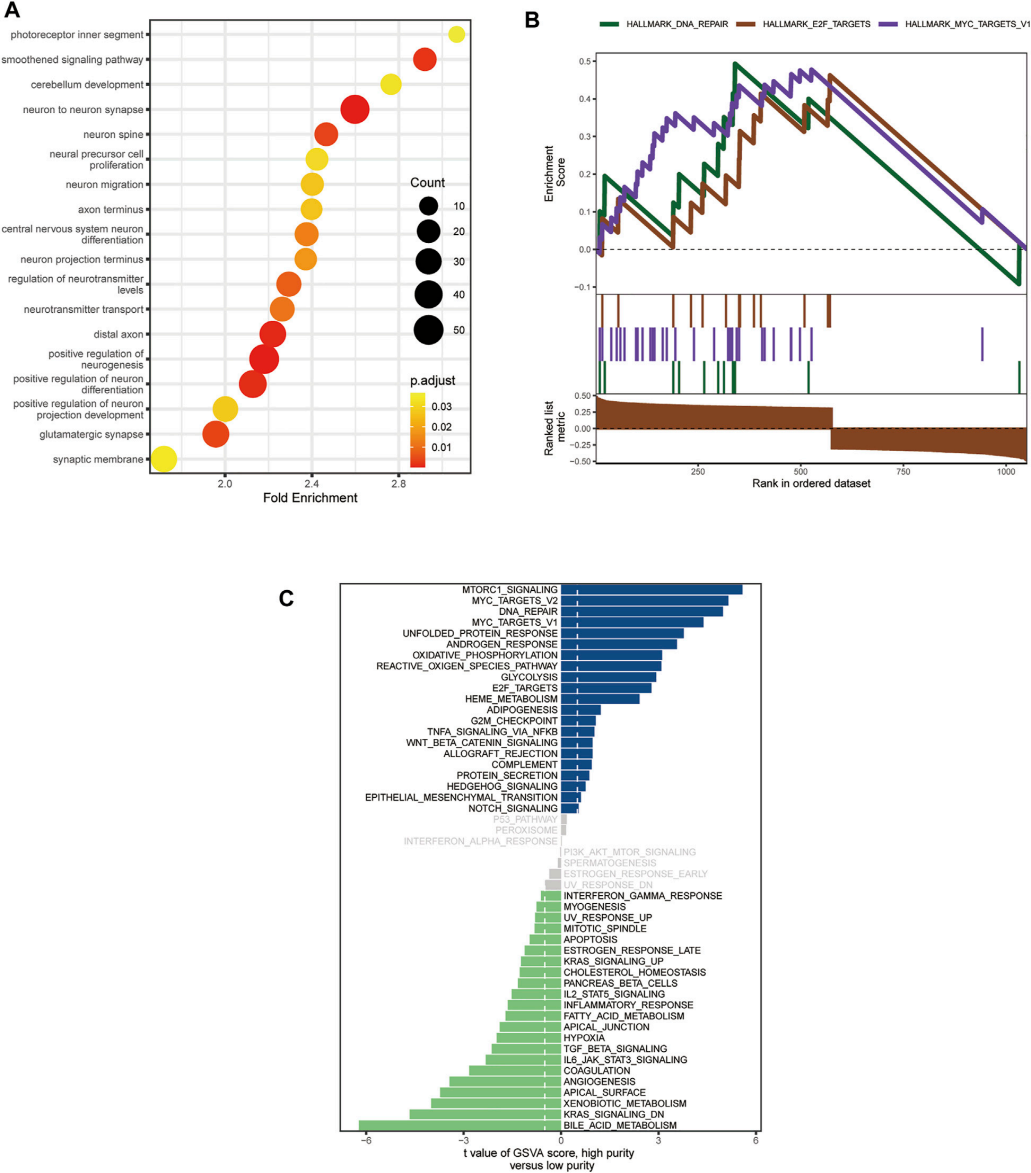
Biological functions related to the purity of medulloblastoma (MB) in the GSE85218 dataset. **(A)** Biological pathways related to the MB purity as revealed by Gene Ontology (GO) analysis; **(B)** biological pathways related to Mb purity as revealed by gene set enrichment analysis (GSEA); **(C)** biological pathways related to MB purity as revealed by gene set variation analysis (GSVA).

Since only the MB samples in the GSE85218 dataset came with gene expression and DNA methylation data as well as complete clinical information, we performed an analysis of tumor purity–related biological functions in this dataset. GO analysis revealed that many development-associated pathways were related to tumor purity (Figure 7A). Gene set enrichment analysis determined the top three biological pathways, including the MYC signaling pathway, DNA repair pathway, and E2F targets signaling pathway (Figure 7B). According to GSVA, the MYC signaling, DNA repair, glycolysis, WNT signaling, Hedgehog signaling, mTORC1 signaling, and oxidative phosphorylation pathways were positively related to tumor purity, whereas the KRAS signaling, IL2-STAT5 signaling, inflammatory response, and angiogenesis pathways were negatively related to tumor purity (Figure 7C).

### Tumor Immune Microenvironment and Tumor Purity

**FIGURE 8:**
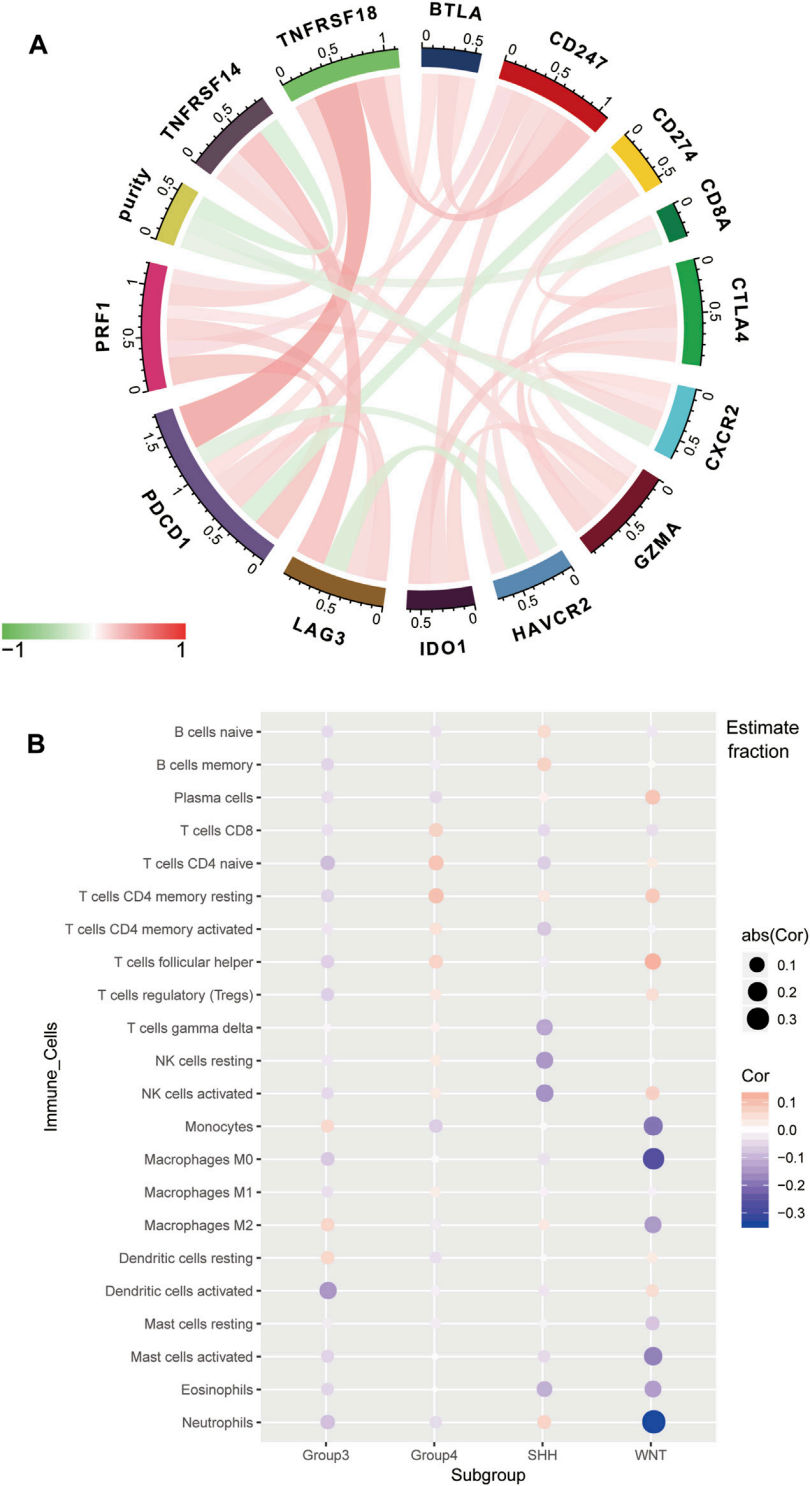
Relationship between the purity of medulloblastoma (MB) and the immune microenvironment in the GSE85218 dataset. **(A)** Relationship between the MB purity and immune-related genes; **(B)** correlation between the purity of each MB subgroup and the CIBERSORT-based infiltrating immunocyte proportions.

**FIGURE 9:**
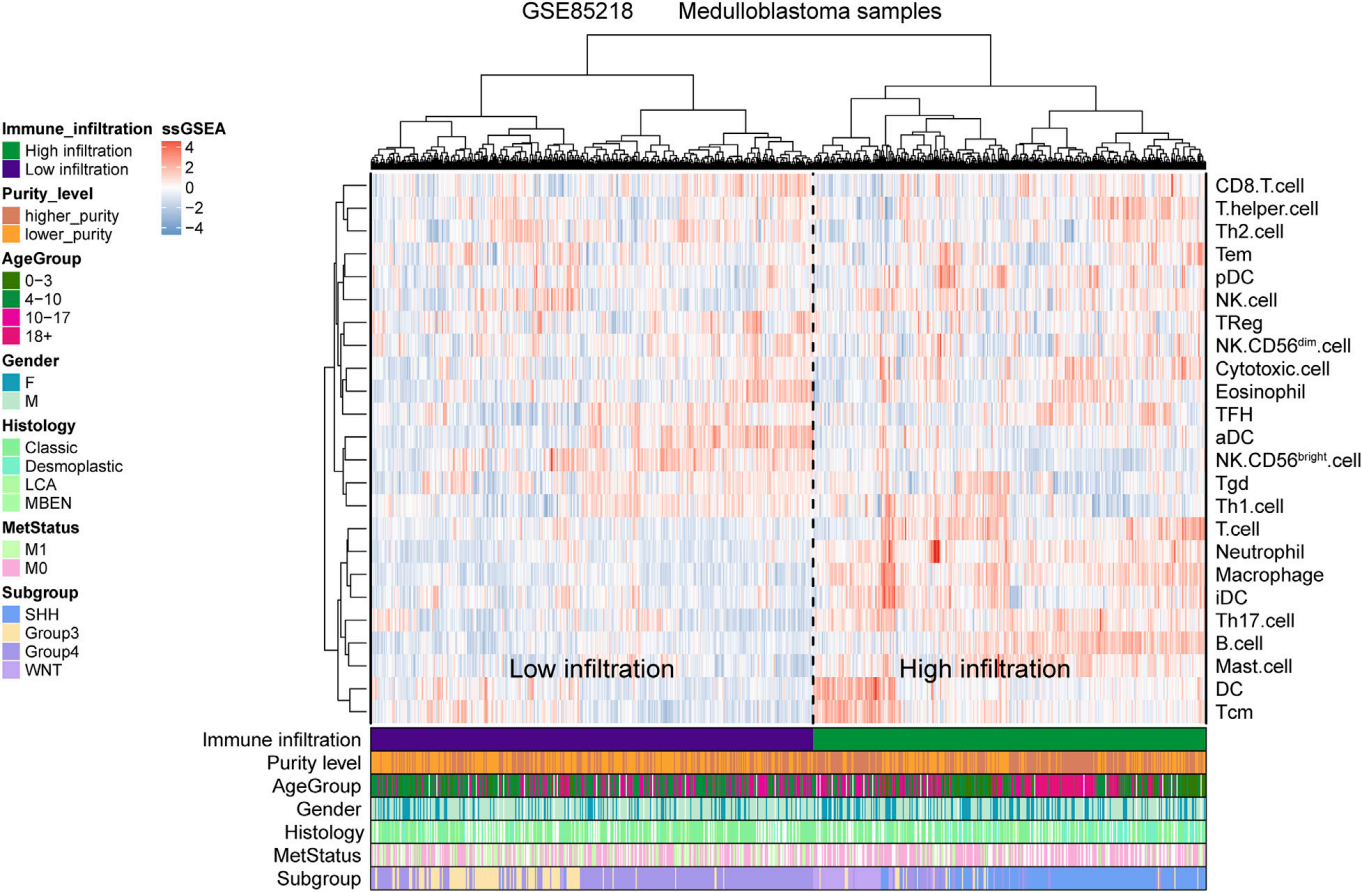
Relationship between the medulloblastoma (MB) purity and the clinical features and immune microenvironment in the GSE85218 dataset as revealed by the single-sample gene set enrichment analysis (ssGSEA).

For the GSE85218 dataset, we also identified the relationship between tumor purity and the immune microenvironment. As indicated in Figure 8A, we found that tumor purity was only negatively related to three immune genes, namely, CD8A (R = −0.18, *p* = 1.06 e-06), CXCR2 (R = −0.18, *p* = 2.90 e-07), and TNFRSF14 (R = −0.21, *p* = 2.58 e-09), but not to other immune-related genes, including the well-known PD1, PD-L1, and CTLA4. Figure 8B reveals the correlation between the tumor purity of each subgroup of MB and CIBERSORT-based proportions of infiltrating immunocytes. In WNT MB, only neutrophils were significantly negatively related to tumor purity (R = 0.34, *p* = 0.004). For SHH MB, only natural killer cells were significantly negatively related to tumor purity (resting, R = −0.14, *p* = 0.03; activated, R = −0.15, *p* = 0.02). However, no statistical correlation was detected between the tumor purity and infiltrating immunocyte proportions in Groups 3 and 4 MB. As shown in Figure 9, WNT and SHH MBs were significantly enriched in the high–immunocyte infiltration group, whereas Groups 3 and 4 MBs were more enriched in the low–immunocyte infiltration group.

### The Prognostic Role of Tumor Purity

**FIGURE 10:**
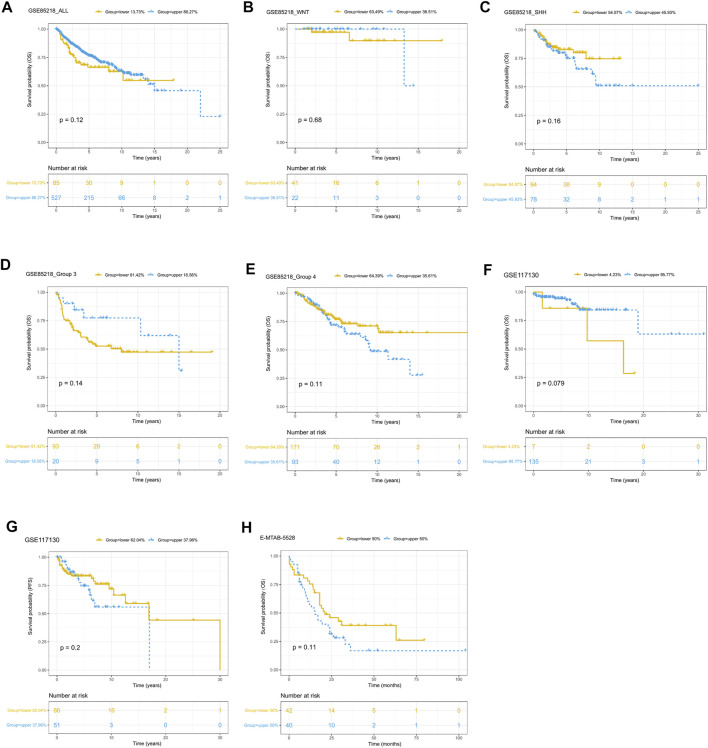
Prognostic role of tumor purity in three pediatric CNS tumor datasets. **(A)** Kaplan–Meier (K-M) curves for overall survival according to tumor purity in the GSE85218 dataset; **(B)** K-M curves for overall survival according to tumor purity of the WNT subgroup medulloblastoma (MB) in the GSE85218 dataset; **(C)** K-M curves for overall survival according to the tumor purity of the SHH subgroup MB in the GSE85218 dataset; **(D)** K-M curves for overall survival according to the tumor purity of Group 3 subgroup MB in the GSE85218 dataset; **(E)** K-M curves for overall survival according to the tumor purity of Group 4 subgroup MB in the GSE85218 dataset; **(F)** K-M curves for overall survival according to the tumor purity in the GSE117130 dataset; **(G)** K-M curves for progression-free survival according to the tumor purity in the GSE117130 dataset; **(H)** K-M curves for overall survival according to the tumor purity in the E-MTAB-5528 dataset.

Since only the GSE85218, GSE117130, and E-MTAB-5528 datasets included clinical outcome data, they were used to assess the relationship between tumor purity and clinical outcome. For each type of pediatric CNS tumor, we divided the patients into a high-purity group and a low-purity group. As shown in Figures 10A–H, the two groups did not differ much in terms of survival rate in all of the CNS tumor datasets. The aforementioned findings suggest that among all pediatric CNS tumors, the association between tumor purity and patient prognosis may be weak.

## Discussion

With the development of high-throughput techniques, many novel computation methods based on bioinformatics could be employed to infer tumor purity. In contrast to those based on histopathology, bioinformatics algorithms elicit more highly concordant and objective results. In this study, we performed a comprehensive purity analysis of pediatric CNS tumors with DNA methylation data and gene expression data from several CNS tumor–related large sample datasets on the basis of three tumor purity calculation methods (namely, RF, InfiniumPurify, and ESTIMATE). We found that only the RF estimation approach could produce normally distributed tumor purity.

These results suggest that 1) to prevent bias arising from the introduction of other tumor molecular data, we should employ high-throughput data of the same tumor type (glioma in this study) to construct a prediction model for estimating tumor purity; and 2) given that the presence of non-immune and stromal cells in CNS tumor tissues may affect the purity estimation results of indirect algorithms such as ESTIMATE, it is more reasonable to choose direct methods of tumor purity estimation. We found that there was some relationship between pediatric CNS tumor purity and the molecular and clinicopathologic features. These findings suggested that tumor purity may be an intrinsic characteristic of pediatric CNS tumors. When analyzing the purity of MB in a systematic way, we discovered that tumor purity was lower in Groups 3 and 4 MBs with a worse prognosis than in WNT and SHH MBs. This is consistent with previous studies with regard to glioma purity (Zhang et al., 2017). A possible reason for this is that Groups 3 and 4 MBs are more inclined to undergo metastasis and tumor cell spreading and have difficulty forming dense solid bulks.

An enrichment analysis of MB purity–related biological pathways unveiled some classical signaling pathways related to the biology of MB, including MYC, WNT, and Hedgehog pathways (Northcott et al., 2011). For instance, the WNT pathway is enriched in WNT MB, and the sonic Hedgehog pathway is enriched in SHH MB (Northcott et al., 2011; Ramaswamy and Taylor, 2017; Wang et al., 2018). Moreover, amplification of the MYC oncogene is the most common genetic alteration of Group 3 MB (Ramaswamy and Taylor, 2017; Wang et al., 2018). In addition, we found that some development-associated pathways were associated with tumor purity; thus, abnormalities in such pathways may lead to the occurrence of MB. In the correlation analysis of MB purity and the immune microenvironment, three genes related to immunity, namely, CD8A, CXCR2, and TNFRSF14, were negatively related to tumor purity. These findings suggested that such immune-related genes may be potential targets for immune microenvironment–specific MB therapies. On the other hand, genes related to classical immunosuppression checkpoints, such as PD-1, PD-L1, and CTLA4, were not significantly associated with MB purity. This finding indicates that the efficacy of immunotherapies with PD-1, PD-L1, and CTLA4 inhibitors may be limited to MB. In addition, most infiltrating immunocytes were unrelated to MB purity, indicating that immunocyte-based therapies may also be limited to MB.

While exploring the tumor purity–related survival analyses of MB, EPN, and pediatric high-grade glioma, we confirmed that the effect of tumor purity was insignificant for the survival of patients. These results are inconsistent with previous studies on tumor purity (Aran et al., 2015; Zhang et al., 2017). Cancer cells are capable of recruiting immune infiltrating cells to the glioma microenvironment (Silver et al., 2016), which could influence the prognosis of glioma patients (Zhang et al., 2017). However, childhood brain tumors are considered to be relatively immunologically “cold” due to the lack of genetic mutations (Gröbner et al., 2018). Furthermore, Bockmayr et al. did not observe associations between intratumoral immune infiltrates and MB survival, and they attributed their results to the overall very low immune infiltration (Bockmayr et al., 2018). The hypothesis that the ability of pediatric CNS tumors to recruit immune infiltrating cells is relatively weak may provide a direction for why tumor purity does not influence the overall survival of pediatric CNS tumor patients. In addition, these results may indirectly confirm the difference between children’s CNS tumors and adults’ brain tumors in terms of clinical and molecular features.

Nevertheless, the present work has some limitations. First, our findings require external validation using independent pediatric CNS tumor datasets. Second, due to the retrospective setting of the present study, additional prospective studies are necessary to evaluate our conclusions.

## Conclusion

We presented a systematic comparison of three tumor purity estimation methods across pediatric CNS tumors and found that the RF algorithm is applicable for pediatric CNS tumor purity estimation. MB purity was significantly associated with some classical signaling pathways associated with MB biology and development-related pathways. Furthermore, our analysis showed a minor effect of tumor purity on the survival of pediatric patients with CNS tumors. It is important for future studies of pediatric CNS tumors to take tumor purity into account when analyzing high-throughput data from patient samples.

## Data Availability

The datasets presented in this study can be found in online repositories. The names of the repository/repositories and accession number(s) can be found in the article/Supplementary Material.

## Abbreviations

CNS, Central nervous system; DEGs, Differentially expressed genes; EPN, Ependymoma; GEO, Gene Expression Omnibus; GO, Gene ontology; GSEA, Gene set enrichment analysis; GSVA, Gene set variation analysis; K-M, Kaplan–Meier; MB, Medulloblastoma; RF, Random forest; ssGSEA, Single-sample gene set enrichment analysis; TME, Tumor microenvironment.
